# Combination therapy of exendin-4 and allogenic adipose-derived mesenchymal stem cell preserved renal function in a chronic kidney disease and sepsis syndrome setting in rats

**DOI:** 10.18632/oncotarget.21727

**Published:** 2017-10-10

**Authors:** Chih-Hung Chen, Ben-Chung Cheng, Kuan-Hung Chen, Pei-Lin Shao, Pei-Hsun Sung, Hsin-Ju Chiang, Chih-Chao Yang, Kun-Chen Lin, Cheuk-Kwan Sun, Jiunn-Jye Sheu, Hsueh-Wen Chang, Mel S. Lee, Hon-Kan Yip

**Affiliations:** ^1^ Division of General Medicine, Department of Internal Medicine, Kaohsiung Chang Gung Memorial Hospital and Chang Gung University College of Medicine, Kaohsiung, 83301, Taiwan; ^2^ Division of Nephrology, Department of Internal Medicine, Kaohsiung Chang Gung Memorial Hospital and Chang Gung University College of Medicine, Kaohsiung, 83301, Taiwan; ^3^ Department of Anesthesiology, Kaohsiung Chang Gung Memorial Hospital and Chang Gung University College of Medicine, Kaohsiung, 83301, Taiwan; ^4^ Department of Nursing, Asia University, Taichung, 41354, Taiwan; ^5^ Division of Cardiology, Department of Internal Medicine, Kaohsiung Chang Gung Memorial Hospital and Chang Gung University College of Medicine, Kaohsiung, 83301, Taiwan; ^6^ Department of Obstetrics and Gynecology, Kaohsiung Chang Gung Memorial Hospital and Chang Gung University College of Medicine, Kaohsiung, 83301, Taiwan; ^7^ Department of Emergency Medicine, E-Da Hospital, I-Shou University School of Medicine for International Students, Kaohsiung, 82445, Taiwan; ^8^ Division of thoracic and Cardiovascular Surgery, Department of Surgery, Kaohsiung Chang Gung Memorial Hospital and Chang Gung University College of Medicine, Kaohsiung, 83301, Taiwan; ^9^ Department of Biological Sciences, National Sun Yat-Sen University, Kaohsiung, 80424, Taiwan; ^10^ Department of Orthopedics, Kaohsiung Chang Gung Memorial Hospital and Chang Gung University College of Medicine, Kaohsiung, 83301, Taiwan; ^11^ Institute for Translational Research in Biomedicine, Kaohsiung Chang Gung Memorial Hospital, Kaohsiung, 83301, Taiwan; ^12^ Center for Shockwave Medicine and Tissue Engineering, Kaohsiung Chang Gung Memorial Hospital, Kaohsiung, 83301, Taiwan; ^13^ Department of Medical Research, China Medical University Hospital, China Medical University, Taichung, 40402, Taiwan

**Keywords:** chronic kidney disease, sepsis syndrome, exendin-4, adipose-derived mesenchymal stem cell, inflammation

## Abstract

Combined therapy with exendin-4 (Ex4) and allogenic adipose-derived mesenchymal stem cells (ADMSC) was tested against either therapy alone for protecting kidney function against chronic kidney disease (CKD) complicated by sepsis syndrome (SS) [i.e., by intraperitoneal injection of cecal-derived bacteria (1.0 × 10^4^) cells/milliliter/total 5.0 cc].Adult-male-Sprague Dawley rats (n=36) were equally divided into group 1 (sham-control), group 2 (CKD), group 3 (CKD-SS), group 4 (CKD-SS-Ex4), group 5 (CKD-SS-ADMSC) and group 6 (CKD-SS-Ex4-ADMSC). At day 42 after CKD induction SS was induced. Thirty-minutes after SS induction, ADMSCs (2.0 ×10^6^ cells) were intravenously administered to groups 5 and 6. Ex4 (10 μg/kg) was intraperitoneally administered groups 4 and 6 at 30 min and days 1 to 5 after SS induction. Animals were euthanized at day 47 after CKD induction. Kidney-injury score, collagen-deposition area, and creatinine/BUN levels were lowest in group 1, highest in group 3 and significantly higher in group 2 than in groups 4 to 6 in a progressively increasing manner (all P<0.0001). Protein expressions of inflammatory (MMP-9/TNF-α/NF-κB/IL-1ß/ICAM-1), oxidative-stress (NOX-1/NOX-2/oxidized protein), apoptotic (mitochondrial-Bax/cleaved-caspase-3/cleaved-PARP) and fibrotic/DNA-damaged (Smad3/TGF-ß/γ-H2AX) biomarkers showed an identical pattern, whereas anti-fibrotic (BMP-2/Smad1/5), anti-apoptotic/endothelial-integrity (Bcl-2/eNOS) and podocyte-integrity (ZO-1/p-cadherin) biomarkers exhibited an opposite pattern of kidney-injury score among the six groups (all P>0.0001). Cellular expressions of inflammatory (CD14/CD68) and glomerulus/tubular-injury (WT-1/KIM-1) biomarkers displayed an identical pattern, whereas glomerulus/podocyte-component (dystroglycan/nephrin/ZO-1/fibronectin/p-cadherin) biomarkers showed an opposite kidney-injury score among the six groups (all P<0.0001). In conclusion, Ex4-ADMSC therapy effectively preserved renal function in the CKD-SS setting.

## INTRODUCTION

Chronic kidney disease (CKD), a current leading public health concern, is becoming a global burden, despite recent advances in management [1]. By definition, CKD is characterized by an irreversible decrease in kidney function. CKD can be associated with a higher risk of progression to end-stage renal disease [2]. Mortality is substantially elevated in patients with CKD, including those with end-stage renal disease, treated with dialysis, or renal transplant recipients [2–9].

Clinical observational studies have established that CKD affects about 10% of the general adult population worldwide, and most cases are complicated by sepsis and cardiovascular disease [10, 11]. Additionally, even after the beginning of replacement therapy in end stage renal failure patients, mortality rate has been estimated to exceed 20% in the first year [12]. Further analysis has shown that more than 50% of these deaths are contributed to by cardiovascular diseases, of which 20% are caused by myocardial infarction [10]. Thus, it is easy to understand that health care costs are enormously high [13, 14].

CKD is not only commonly found to coexist with cardiovascular disease [10, 11], but is also found to be more frequently affected by infection/sepsis, perhaps due to intrinsic prosperity of immunocompromised matter [10, 11]. The in-hospital mortality rate in the setting of sepsis has been reported to be unacceptably high in acute kidney injury (AKI) patients with and without preexisting CKD [15–18]. Of importance, even when CKD patients survive from sepsis, the post-sepsis prognostic outcome of these patients is much poorer [17] as compared with those of non-sepsis matched controls [11] mainly due to significantly increased long-term risk of cardiovascular events [11] and further significant loss of residual renal function which is a strongly independent predictor of poor prognostic outcome in CKD patients [19, 20]. Accordingly, how to preserve the residual renal function in sepsis CKD patients is of utmost importance for improving post-sepsis long-term survival.

Exendin-4 (Ex4), a glucagon-like peptide-1 (GLP-1) analogue, has been identified to have multi-organ protective effects mainly through several mechanistic pathways, namely, anti-oxidative, anti-inflammatory, anti-fibrotic and anti-apoptotic [21–23]. Additionally, many studies have demonstrated that mesenchymal stem cells, especially those of adipose-derived mesenchymal stem cells (ADMSCs), possess not only the intrinsic capacity of immunomodulation, but can also attenuate inflammation and immune responses [24–28]. Based on these considerations [10-20, 24-28], by using an experimental model of CKD-complicated sepsis syndrome (SS), in this study we tested the hypothesis that combined therapy with exendin-4 and ADMSCs was superior to either one alone for protecting renal function against CKD complicated by SS.

## RESULTS

### Serial changes of circulating levels of blood urea nitrogen (BUN) and creatinine, ratio of urine protein to creatinine and kidney injury score at day 47 after CKD induction (Figure 1)

Prior to CKD induction, the circulating levels of BUN and creatinine and the ratio of urine protein to creatinine did not differ among the six groups. However, by day 35 after CKD induction, these three parameters were significantly lower in group 1 sham-control (SC) than in the other five groups but they showed no difference among these latter five groups. Additionally, by day 47 after CKD induction, these three parameters were highest in group 3 (CKD-SS), lowest in group 1, significantly higher in group 2 (CKD) than in groups 4 (CDK-SS + Ex4), 5 (CKD-SS + ADMSC) and 6 (CKD-SS + Ex4 + ADMSC), and significantly higher in groups 4 and 5 than in group 6, but they showed no difference between groups 4 and 5.

**Figure 1:**
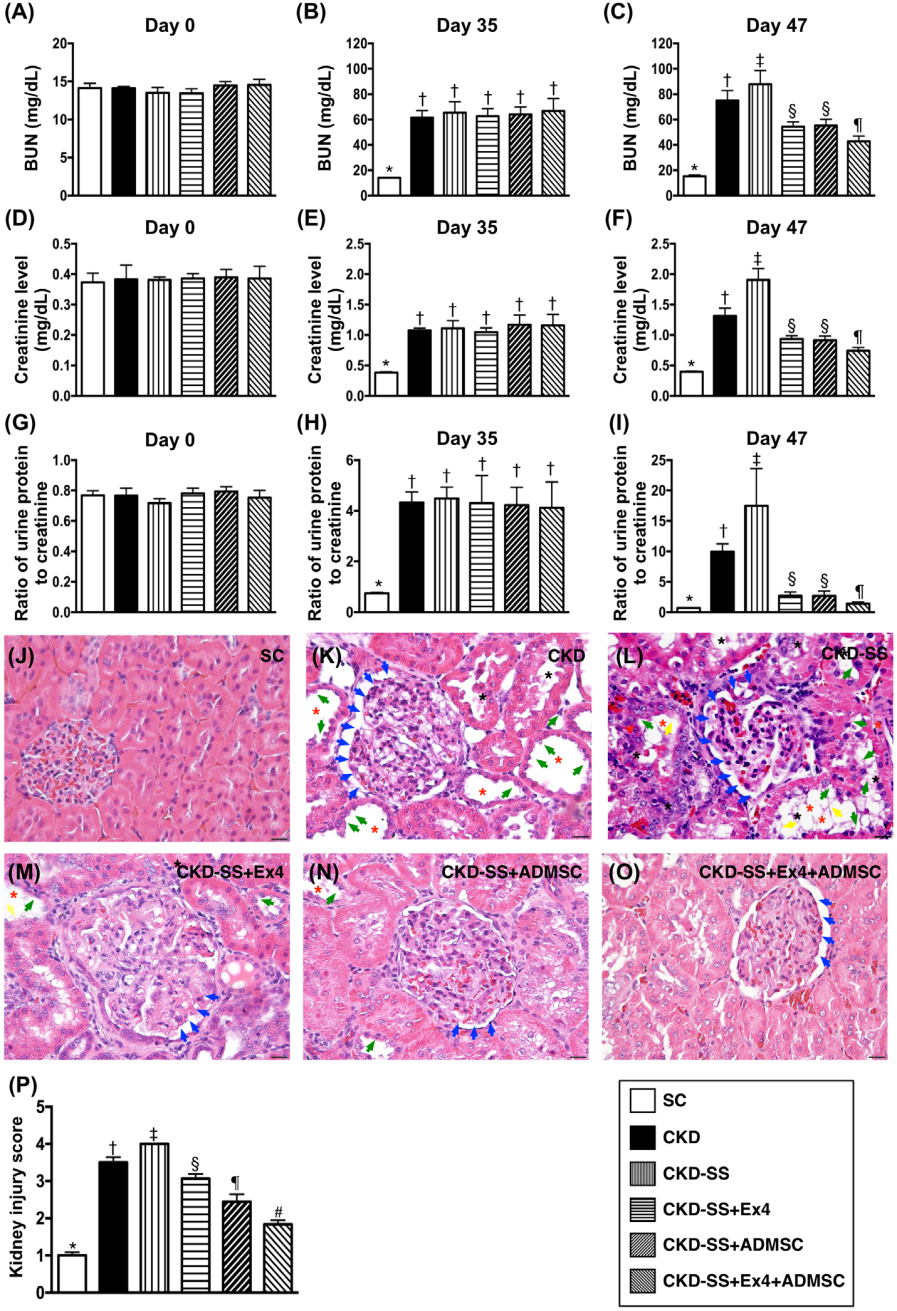
Time courses of circulating BUN and creatinine levels and ratio of urine protein to creatinine, and kidney injury score at day 47 after CKD induction **(A)** At day 0: blood urea nitrogen (BUN) level, p>0.5. **(B)** At day 35, BUN level,^*^ vs. †, p<0.0001. **(C)** At day 47, BUN level, ^*^ vs. other groups with different symbols (†, ‡, §, ¶), p<0.0001. **(D)** At day 0: creatinine level, p>0.5. **(E)** At day 35, creatinine level, ^*^ vs. †, p<0.0001. **(F)** At day 47, creatinine level, ^*^ vs. other groups with different symbols (†, ‡, §, ¶), p<0.0001. **(G)** At day 0: ratio of urine protein to creatinine, p>0.5. **(H)** At day 35, ration of urine protein to creatinine, ^*^ vs. †, p<0.0001. **(I)** At day 47, ratio of urine protein to creatinine, ^*^ vs. other groups with different symbols (†, ‡, §, ¶), p<0.0001. **(J** to **O)** Light microscopic findings of H. & E. stain (400x) demonstrating significantly higher degree of loss of brush border in renal tubules (yellow arrows), tubular necrosis (green arrows), tubular dilatation (red asterisk) protein cast formation (black asterisk), and dilatation of Bowman’s capsule (blue arrows) in CKD and CKD-SS group than in other groups. Scale bars in right lower corner represent 20μm. **(P)** Analytical result of kidney injury score by day 47, ^*^ vs. other groups with different symbols (†, ‡, §, ¶, ^#^), p<0.0001. All statistical analyses were performed by one-way ANOVA, followed by Bonferroni multiple comparison post hoc test (n=6 for each group). Symbols (^*^, †, ‡, §, ¶, ^#^) indicate significance (at 0.05 level). SC = sham control; CKD = chronic kidney disease; SS = sepsis syndrome; ADMSC = adipose derive mesenchymal stem cell; Ex4 = exendin 4.

The kidney injury score induction showed a comparable pattern of creatinine level at day 47 after CKD induction except that this parameter was significantly higher in group 4 than in group 5.

### Protein expressions of inflammatory reaction by day 47 after CKD induction (Figure 2)

The protein expression of matrix metalloproteinase (MMP)-9 and tumor necrosis factor (TNF)-α, two indicators of inflammatory biomarkers, were highest in group 3, lowest in group 1, significantly higher in group 2 than in groups 4 to 6 and significantly higher in groups 4 and 5 than in group 6, but they showed no difference between groups 4 and 5. The protein expressions of nuclear factor (NF)-κB, interleukin (IL)-1ß, and intercellular adhesion molecule (ICAM)-1, another four indices of inflammatory biomarkers, exhibited a similar pattern of MMP-9, except that these four parameters were significantly higher in group 4 than in group 5.

**Figure 2:**
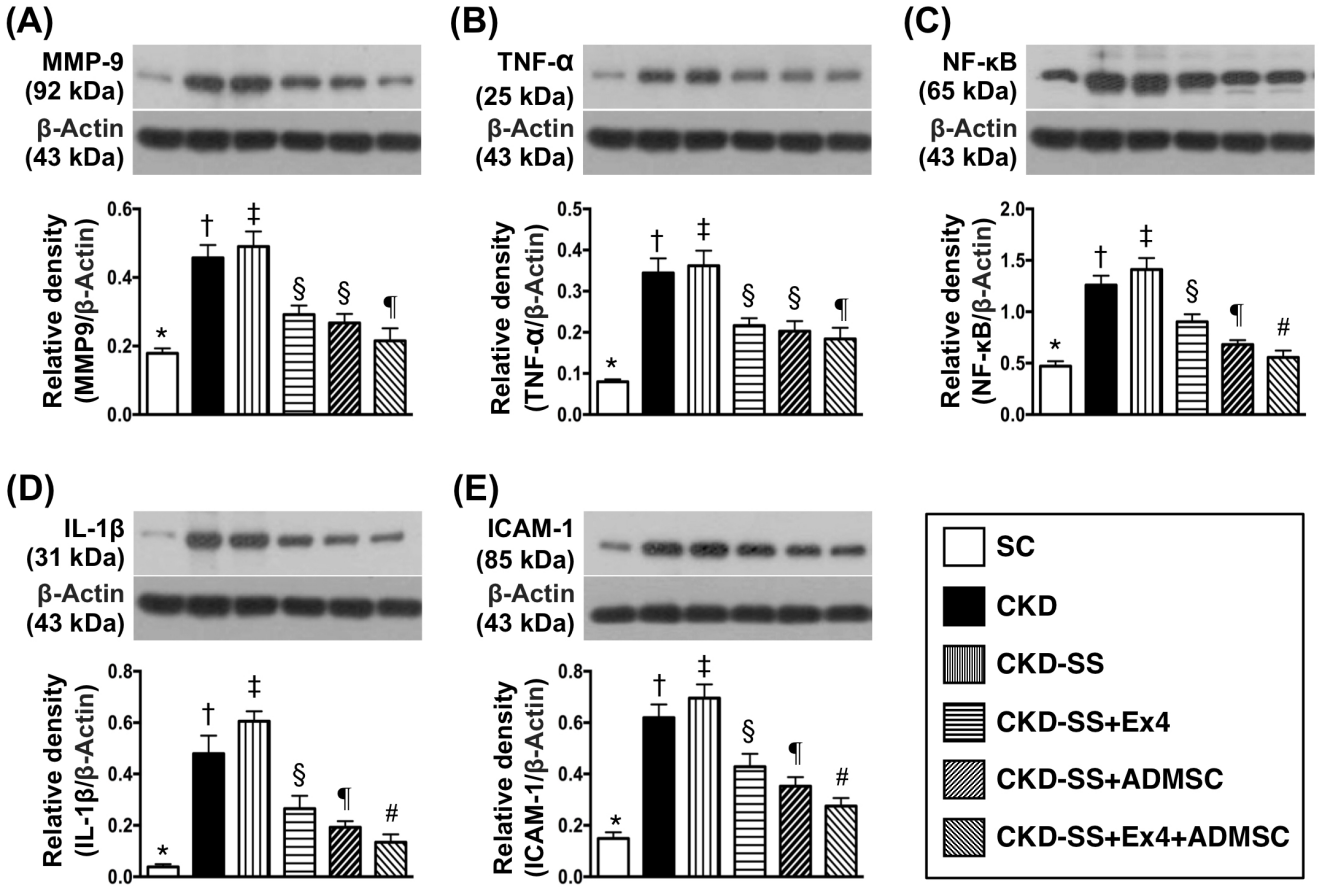
Protein expressions of inflammatory biomarkers in kidney parenchyma at day 47 after CKD induction **(A)** Protein expression of matrix metalloproteinase (MMP)-9, ^*^ vs. other groups with different symbols (†, ‡, §, ¶), p<0.0001. **(B)** Protein expression of tumor necrosis factor (TNF)- α, ^*^ vs. other groups with different symbols (†, ‡, §, ¶), p<0.0001. **(C)** Protein expression of nuclear factor (NF)-κB, ^*^ vs. other groups with different symbols (†, ‡, §, ¶, ^#^), p<0.0001. **(D)** Protein expression of interleukin (IL)-1ß, ^*^ vs. other groups with different symbols (†, ‡, §, ¶, ^#^), p<0.0001. **(E)** Protein expression of intercellular adhesion molecular molecule (ICAM)-1, ^*^ vs. other groups with different symbols (†, ‡, §, ¶, ^#^), p<0.0001. All statistical analyses were performed by one-way ANOVA, followed by Bonferroni multiple comparison post hoc test (n=6 for each group). Symbols (^*^, †, ‡, §, ¶, ^#^) indicate significance (at 0.05 level). SC = sham control; CKD = chronic kidney disease; SS = sepsis syndrome; ADMSC = adipose derive mesenchymal stem cell; Ex4 = exendin 4.

### Protein expressions of oxidative stress biomarkers by day 47 after CKD induction (Figure 3)

The protein expressions of NADPH oxidase (NOX)-1 and NOX-2, two indices of oxidative stress, were highest in group 3, lowest in group 1, significantly higher in group 2 than in groups 4 to 6, significantly higher in groups 4 and 5 than in group 6, and significantly higher in group 4 than in group 5. Additionally, the expression of oxidized protein, another indicator of oxidative stress, was highest in groups 2 and 3, lowest in group 1, and significantly higher in groups 4 and 5 than in group 6, but this parameter exhibited no difference between groups 2 and 3 or between groups 4 and 5.

**Figure 3:**
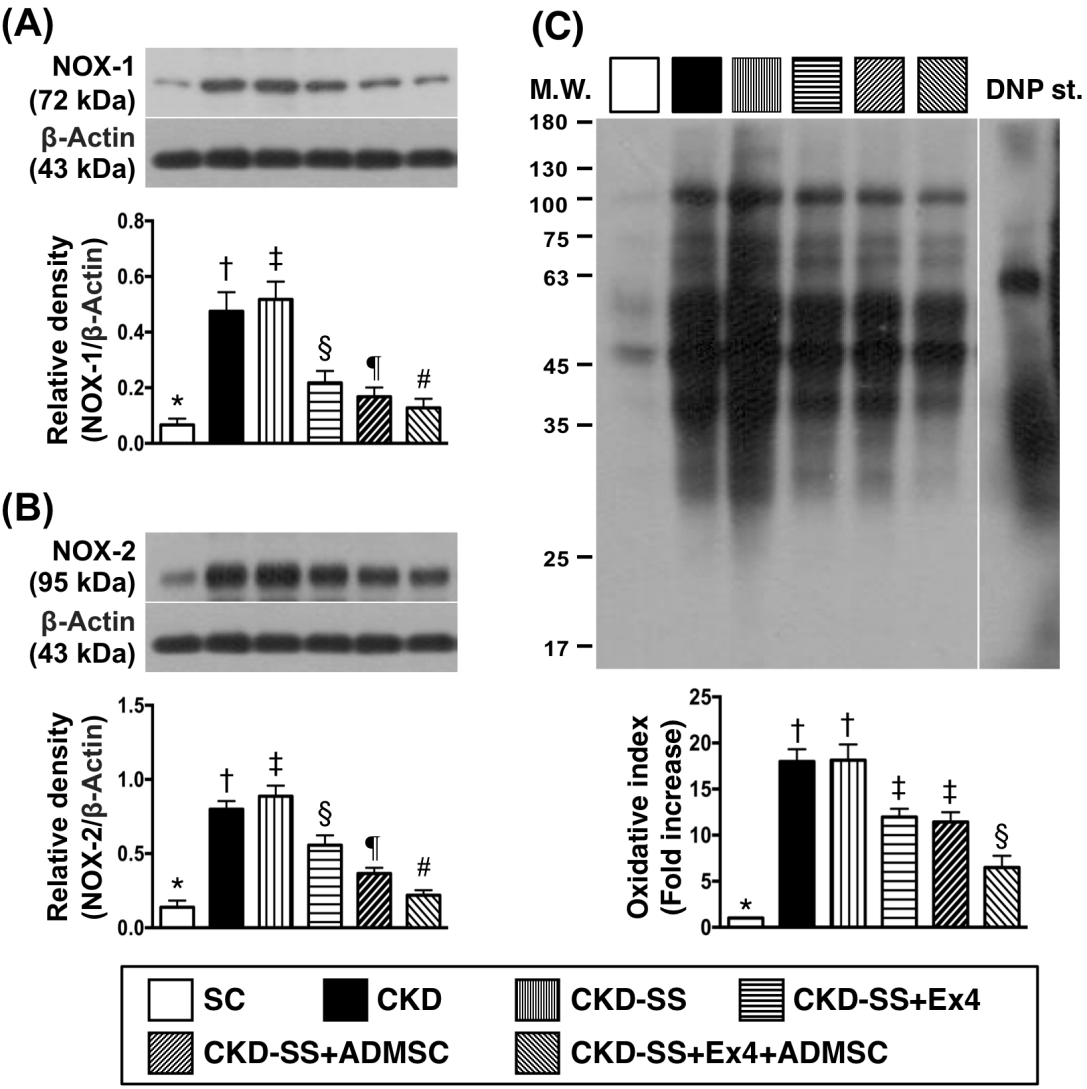
Protein expressions of oxidative stress biomarkers in kidney parenchyma by day 47 after CKD induction **(A)** Protein expression of NOX-1, ^*^ vs. other groups with different symbols (†, ‡, §, ¶, ^#^), p<0.0001. **(B)** Protein expression of NOX-2, ^*^ vs. other groups with different symbols (†, ‡, §, ¶, ^#^), p<0.0001. **(C)** Oxidized protein expression, ^*^ vs. other groups with different symbols (†, ‡, §, ¶, ^#^), p<0.0001. (Note: left and right lanes shown on the upper panel represent protein molecular weight marker and control oxidized molecular protein standard, respectively). M.W = molecular weight; DNP = 1-3 dinitrophenylhydrazone. All statistical analyses were performed by one-way ANOVA, followed by Bonferroni multiple comparison post hoc test (n=6 for each group). Symbols (^*^, †, ‡, §, ¶, ^#^) indicate significance (at 0.05 level). SC = sham control; CKD = chronic kidney disease; SS = sepsis syndrome; ADMSC = adipose derive mesenchymal stem cell; Ex4 = exendin 4.

### Protein expressions of apoptotic, anti-apoptotic and endothelial integrity biomarkers at day 47 after CKD induction (Figure 4)

The protein expression of mitochondrial Bax, cleaved caspase 3 and cleaved poly (ADP-ribose) polymerase (PARP), three indicators of apoptosis, were highest in group 3, lowest in group 1, significantly higher in group 2 than in groups 4 to 6, significantly higher in groups 4 and 5 than in group 6, and significantly higher in group 4 than in group 5. On the other hand, the protein expression of Bcl-2, an indicator of anti-apoptosis and endothelial nitric oxide synthase (eNOS), an indicator of endothelial function integrity, displayed an opposite pattern of apoptosis among the six groups.

**Figure 4:**
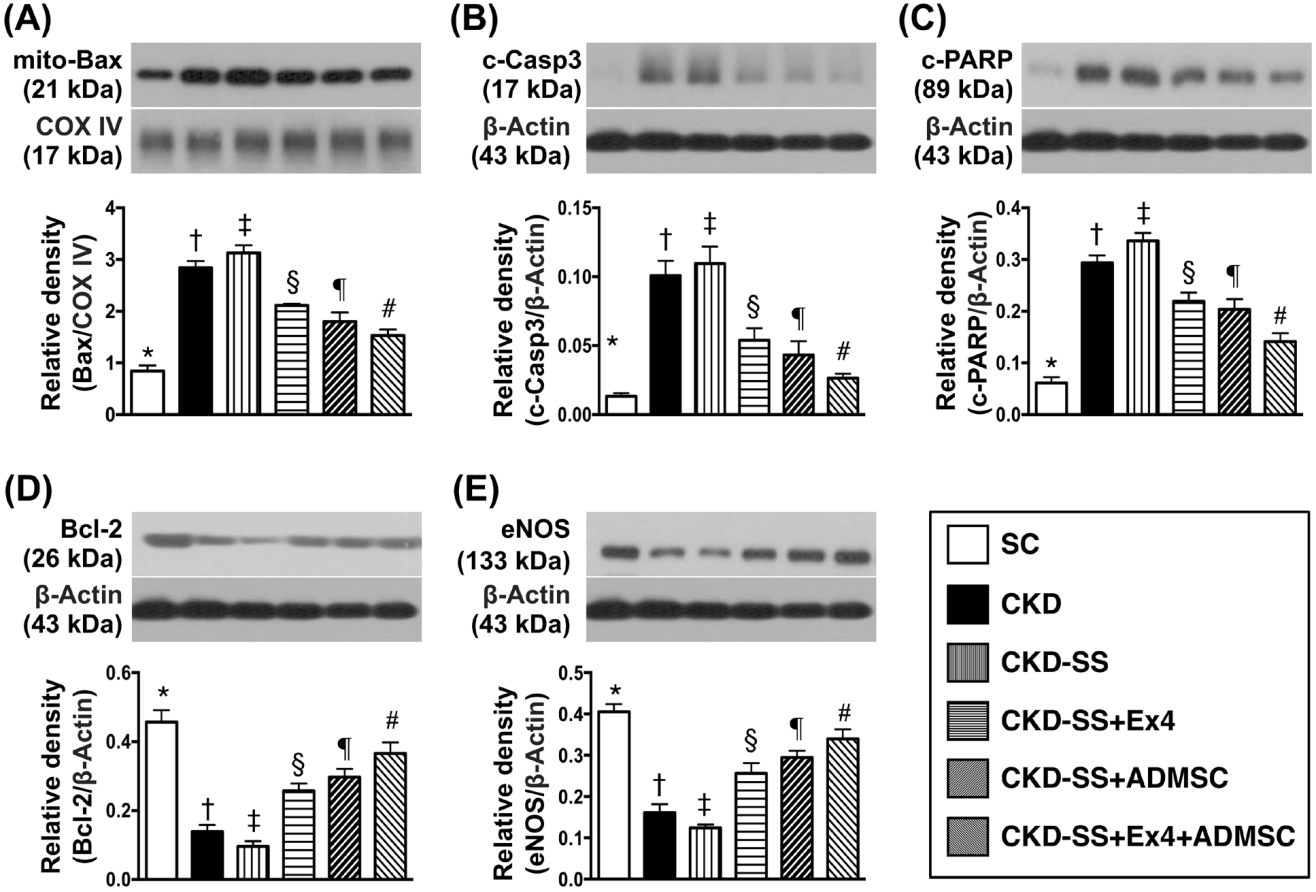
Protein expressions of apoptotic, anti-apoptotic and endothelial integrity in kidney parenchyma biomarkers at day 47 after CKD induction **(A)** Protein expression of mitochondrial (mito)-Bax, ^*^ vs. other groups with different symbols (†, ‡, §, ¶, ^#^), p<0.0001. **(B)** Protein expression of cleaved caspase (c-Casp)-3, ^*^ vs. other groups with different symbols (†, ‡, §, ¶, ^#^), p<0.0001. **(C)** protein expression of cleaved poly (ADP-ribose) polymerase (c-PARP), ^*^ vs. other groups with different symbols (†, ‡, §, ¶, ^#^), p<0.0001. **(D)** Protein expression of Bcl-2, ^*^ vs. other groups with different symbols (†, ‡, §, ¶, ^#^), p<0.0001. **(E)** Protein expression of endothelial nitric oxide synthase (eNOS), ^*^ vs. other groups with different symbols (†, ‡, §, ¶, ^#^), p<0.0001. All statistical analyses were performed by one-way ANOVA, followed by Bonferroni multiple comparison post hoc test (n=6 for each group). Symbols (^*^, †, ‡, §, ¶, ^#^) indicate significance (at 0.05 level). SC = sham control; CKD = chronic kidney disease; SS = sepsis syndrome; ADMSC = adipose derive mesenchymal stem cell; Ex4 = exendin 4.

### Protein expressions of fibrotic, anti-fibrotic and DNA-damaged biomarkers by day 47 after CKD induction (Figure 5)

The protein expressions of Smad3 and transforming growth factor (TGF)-β, two indices of fibrosis were highest in group 3, lowest in group 1, significantly higher in group 2 than in groups 4 to 6, significantly higher in groups 4 and 5 than in group 6, and significantly higher in group 4 than in group 5. Additionally, the protein expression of phosphorylated histone H2AX (γH2AX), a DNA-damage biomarker, showed an identical pattern of fibrosis among the six groups. On the other hand, the protein expressions of Smad1/5 and bone morphogenetic protein-2 (BMP-2), two indicators of anti-fibrosis showed an opposite pattern of fibrosis among the six groups. Additionally, the protein expressions of zonula occludens-1 (ZO-1) and p-cadherin, two indicators of podocyte components, showed an identical pattern of anti-fibrosis among the six groups. Furthermore, the protein expression of E-cadherin, predominantly in renal tubular epithelial cells, displayed an identical pattern of ZO-1, except that this parameter was similar between groups 4 and 5.

**Figure 5:**
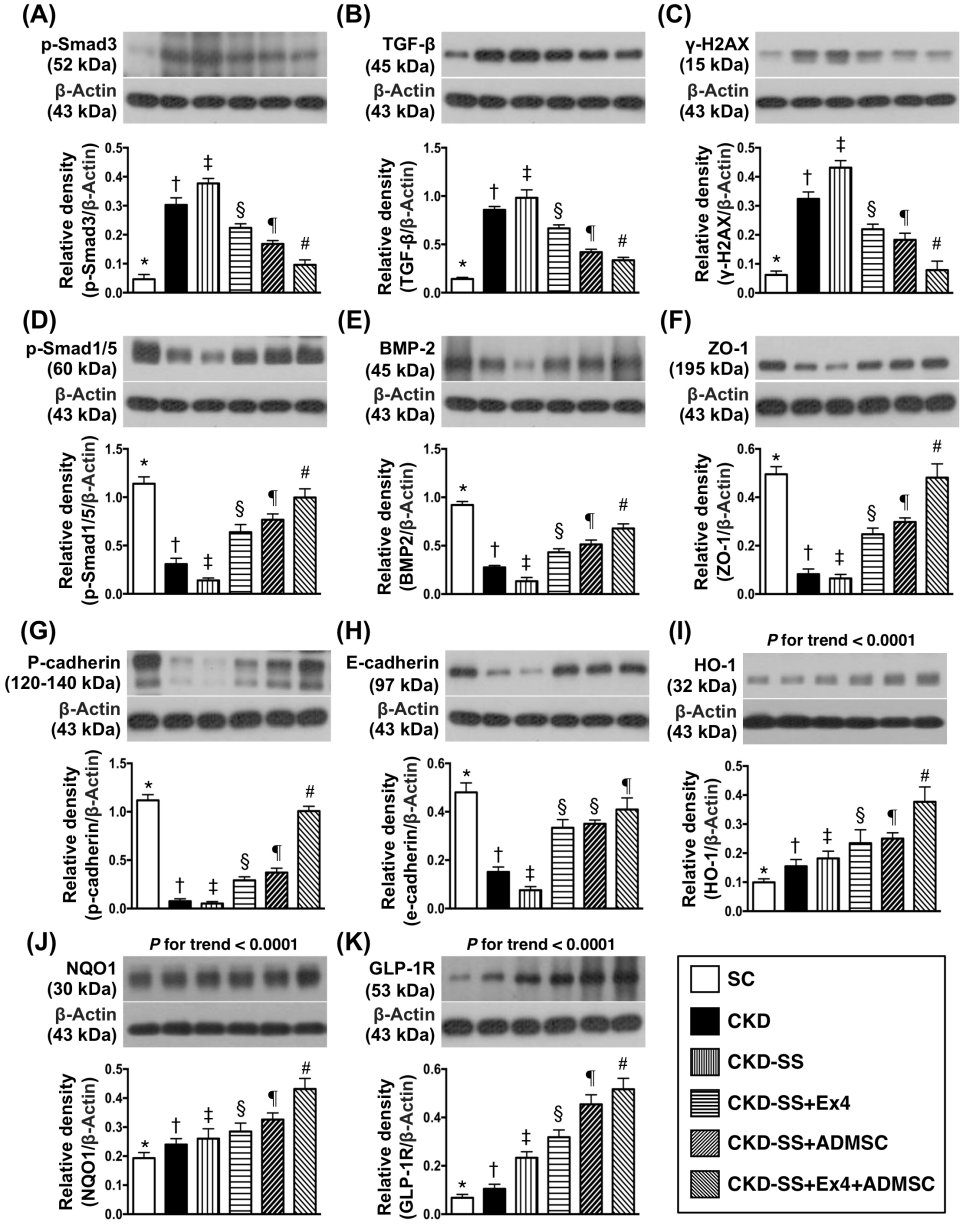
Protein expressions of fibrotic, anti-fibrotic and DNA-damaged biomarkers in kidney parenchyma by day 47 after CKD induction **(A)** Protein expression of Smad3, ^*^ vs. other groups with different symbols (†, ‡, §, ¶, ^#^), p<0.0001. **(B)** Protein expression of transforming growth factor (TGF)-ß, ^*^ vs. other groups with different symbols (†, ‡, §, ¶, ^#^), p<0.0001. **(C)** Protein expression of γ-H2AX, ^*^ vs. other groups with different symbols (†, ‡, §, ¶, ^#^), p<0.0001. **(D)** Protein expression of Smad1/5, ^*^ vs. other groups with different symbols (†, ‡, §, ¶, ^#^), p<0.0001. **(E)** Protein expression of bone morphogenetic protein (BMP)-2, ^*^ vs. other groups with different symbols (†, ‡, §, ¶, ^#^), p<0.0001. **(F)** Protein expression of zonula occludens-1 (ZO-1), ^*^ vs. other groups with different symbols (†, ‡, §, ¶, ^#^), p<0.0001. **(G)** Protein expression of p-cadherin, ^*^ vs. other groups with different symbols (†, ‡, §, ¶, ^#^), p<0.0001. **(H)** Protein expression of E-cadherin, ^*^ vs. other groups with different symbols (†, ‡, §, ¶), p<0.0001. **(I)** Protein expression of heme oxygenase (HO)-1, ^*^ vs. other groups with different symbols (†, ‡, §, ¶, ^#^), p<0.0001. **(J)** Protein expression of NAD (P) H quinone dehydrogenase (NQO1), ^*^ vs. other groups with different symbols (†, ‡, §, ¶, ^#^), p<0.0001. **(K)** Protein expression of Glucagon like peptide 1 receptor (GLP-1R), ^*^ vs. other groups with different symbols (†, ‡, §, ¶, ^#^), p<0.0001. All statistical analyses were performed by one-way ANOVA, followed by Bonferroni multiple comparison post hoc test (n=6 for each group). Symbols (^*^, †, ‡, §, ¶, ^#^) indicate significance (at 0.05 level). SC = sham control; CKD = chronic kidney disease; SS = sepsis syndrome; ADMSC = adipose derive mesenchymal stem cell; Ex4 = exendin 4.

The protein expression of heme oxygenase (HO)-1 and NAD (P) H quinone dehydrogenase (NQO1), two indicators of anti-oxidants, and glucagon like peptide 1 receptor (GLP-1R) were significantly progressively increased from group 1 to 6, suggesting an intrinsic response to ischemic/infectious stimulation and to be further enhanced after exendin-4 and ADMSC treatment.

### Cellular expression of inflammatory biomarkers at day 47 after CKD induction (Figure 6)

Immunofluorescence (IF) microscopy showed that the cellular expression of CD14 and CD68, two indicators of inflammatory cells, were highest in group 3, lowest in group 1, significantly higher in group 2 than in groups 4 to 6, significantly higher in groups 4 and 5 than in group 6, and significantly higher in group 4 than in group 5.

**Figure 6:**
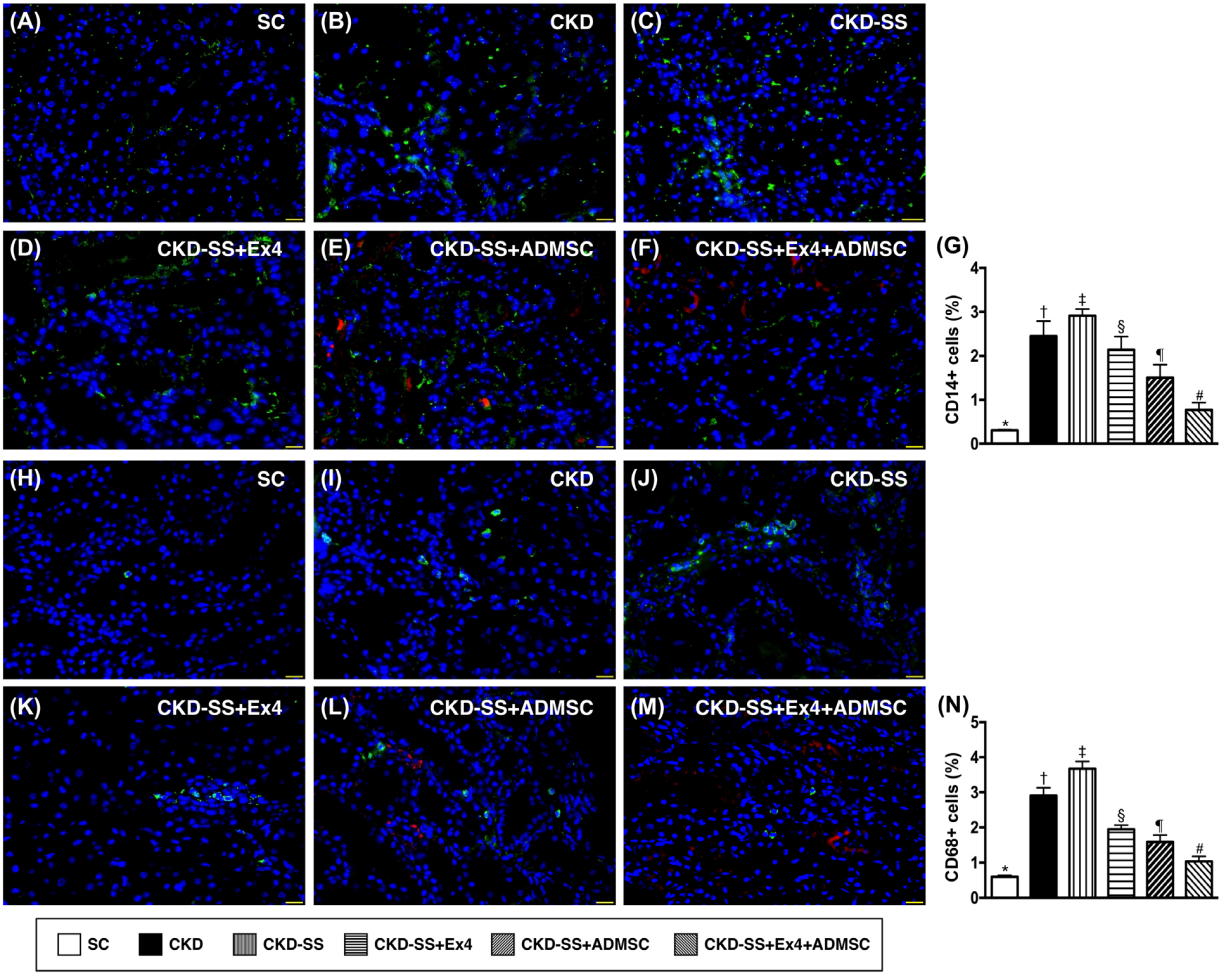
Cellular expression of inflammatory biomarkers in kidney parenchyma at day 47 after CKD induction **(A** to **F)** Illustrating immunofluorescent (IF) microscopic finding (400x) of CD14+ cells (green color). Nuclei were stained by DAPI (blue color). Red color in (E) and (F) indicated ADMSCs were found in kidney parenchyma. **(G)** Analytical result of percentage of CD14+ cells among the four groups, ^*^ vs. other groups with different symbols (†, ‡, §, ¶, ^#^), p<0.0001. **(H** to **M)** Illustrating IF microscopic finding (400x) of CD68+ cells (green color). Nuclei were stained by DAPI (blue color). Red color in (L) and (M) indicated ADMSCs were found in kidney parenchyma. **(N)** Analytical result of percentage of CD68+ cells among the four groups, ^*^ vs. other groups with different symbols (†, ‡, §, ¶, ^#^), p<0.0001. Scale bars in right lower corner represent 20μm. All statistical analyses were performed by one-way ANOVA, followed by Bonferroni multiple comparison post hoc test (n=6 for each group). Symbols (^*^, †, ‡, §, ¶, ^#^) indicate significance (at 0.05 level). SC = sham control; CKD = chronic kidney disease; SS = sepsis syndrome; ADMSC = adipose derive mesenchymal stem cell; Ex4 = exendin 4.

### Microscopy findings of collagen deposition area and cellular expression of Wilm’s tumor suppressor gene 1 (WT-1) at day 47 after CKD induction (Figure 7)

The microscopy finding of Sirius red stain showed that the collagen deposition area, an indicator of tissue/organ fibrosis, was highest in group 3, lowest in group 1, significantly higher in group 2 than in groups 4 to 6, significantly higher in groups 4 and 5 than in group 6, and significantly higher in group 4 than in group 5. Consistently, the expression of WT-1, predominantly in podocytes, showed an identical pattern of collagen deposition among the six groups.

**Figure 7:**
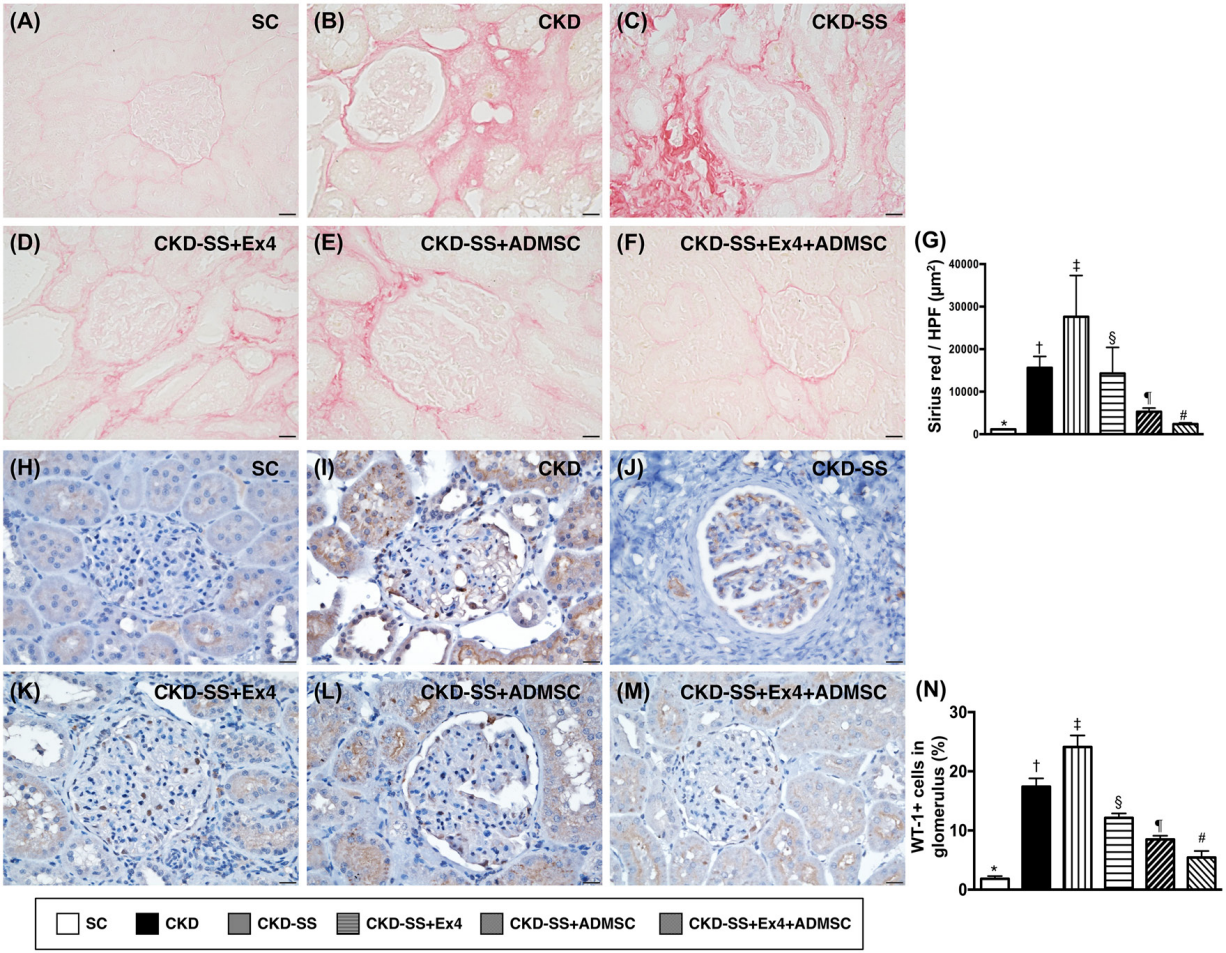
Microscopic findings of collagen deposition area and cellular expression of WT-1 in kidney parenchyma at day 47 after CKD induction **(A** to **F)** Illustrating microscopic finding of Sirius red stain microscopy (400x) for identification of collagen deposition (pink color). **(G)** Analytical result of the collagen deposition area, ^*^ vs. other groups with different symbols (†, ‡, §, ¶, ^#^), p<0.0001. **(H** to **M)** Illustrating microscopic finding (400x) of immunohistochemical staining for identification of Wilm's tumor suppressor gene 1 (WT-1) expressed predominantly in podocyte (brown color). **(N)** Analytical results of WT-1 expression, ^*^ vs. other groups with different symbols (†, ‡, §, ¶, ^#^), p<0.0001. Scale bars in right lower corner represent 20μm. All statistical analyses were performed by one-way ANOVA, followed by Bonferroni multiple comparison post hoc test (n=6 for each group). Symbols (^*^, †, ‡, §, ¶, ^#^) indicate significance (at 0.05 level). SC = sham control; CKD = chronic kidney disease; SS = sepsis syndrome; ADMSC = adipose derive mesenchymal stem cell; Ex4 = exendin 4.

### Cellular expression of ZO-1 and fibronectin at day 47 after CKD induction (Figure 8)

IF showed that the expression of ZO-1, a tight junction-associated protein which provides a link between the integral membrane proteins and the filamentous cytoskeleton in podocytes, was highest in group 1, lowest in group 3, significantly lower in group 2 than in groups 4 to 6, significantly lower in groups 4 and 5 than in group 6, and significantly lower in group 4 than in group 5. Additionally, the IF showed that the amount of fibronectin, predominantly in proximal renal tubules, displayed an identical pattern to ZO-1 in the six groups.

**Figure 8:**
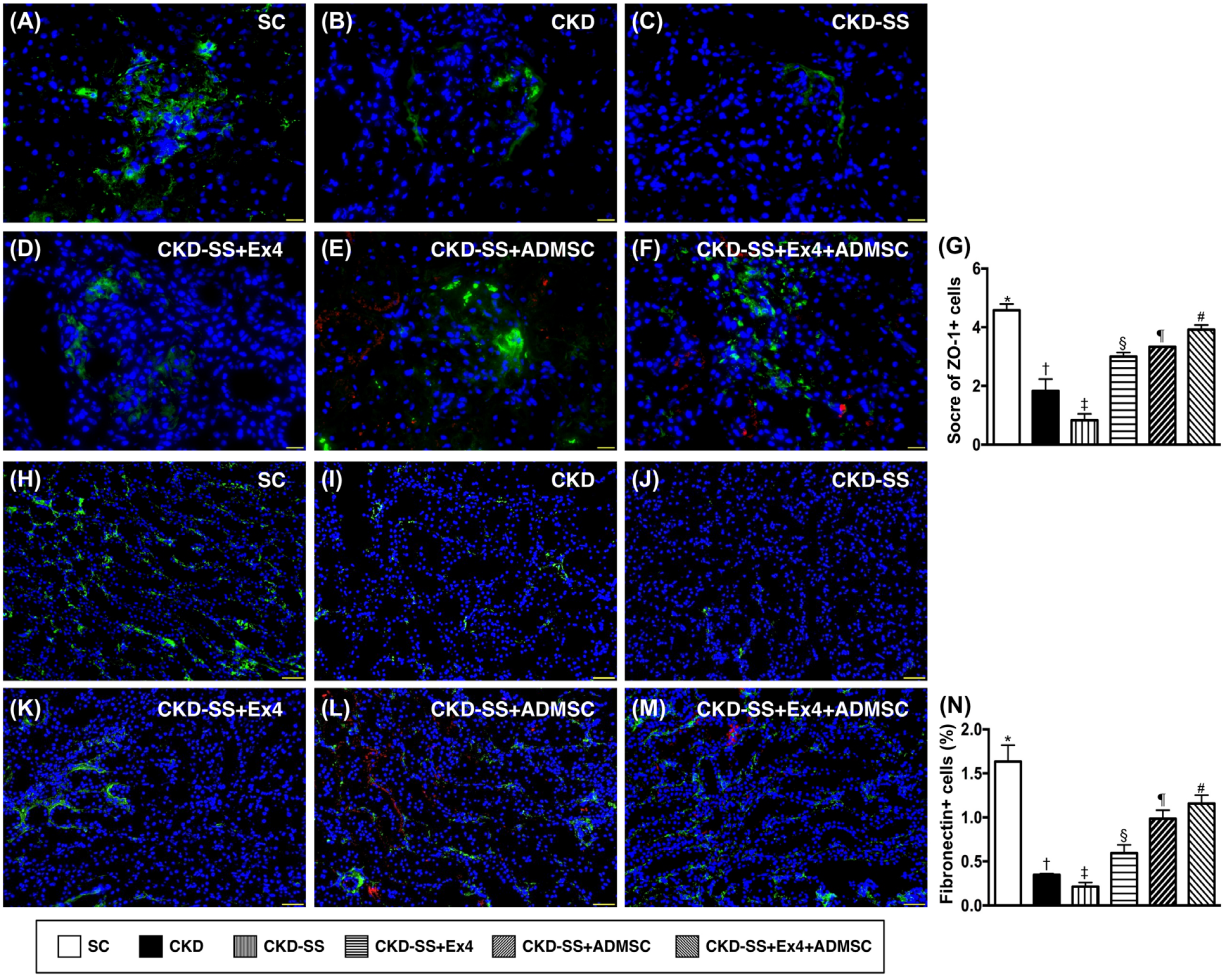
Cellular expression of ZO-1 and fibronectin in kidney parenchyma at day 47 after CKD induction **(A** to **F)** Immunofluorescent (IF) microscopic finding (400x) for identification of zonula occludens-1 (ZO-1) (green color) in the renal glomerulus. Red color in (E) and (F) indicated ADMSCs were found in kidney parenchyma. Scale bars in right lower corner represent 20μm. **(G)** Analytical results of ZO-1 expression, ^*^ vs. other groups with different symbols (†, ‡, §, ¶, ^#^), p<0.0001. **(H** to **M)** IF microscopic finding (200x) for identification of fibronectin in the glomerulus (green color). Red color in (L) and (M) indicated ADMSCs were found in kidney parenchyma. **(N)** Analytical results of fibronectin expression, ^*^ vs. other groups with different symbols (†, ‡, §, ¶, ^#^), p<0.0001. Nuclei were stained by DAPI (blue color). Scale bars in right lower corner represent 50μm. All statistical analyses were performed by one-way ANOVA, followed by Bonferroni multiple comparison post hoc test (n=6 for each group). Symbols (^*^, †, ‡, §, ¶, ^#^) indicate significance (at 0.05 level). SC = sham control; CKD = chronic kidney disease; SS = sepsis syndrome; ADMSC = adipose derive mesenchymal stem cell; Ex4 = exendin 4.

### Cellular expression of P-cadherin and E-cadherin at day 47 after CKD induction (Figure 9)

Immunohistochemical (IHC) analysis showed that the expressions of P-cadherin (predominantly in glomeruli) and E-cadherin were (situated predominantly in renal tubular epithelial cells), were highest in group 1, lowest in group 3, significantly lower in group 2 than in groups 4 to 6, significantly lower in groups 4 and 5 than in group 6, and significantly lower in group 4 than in group 5.

**Figure 9:**
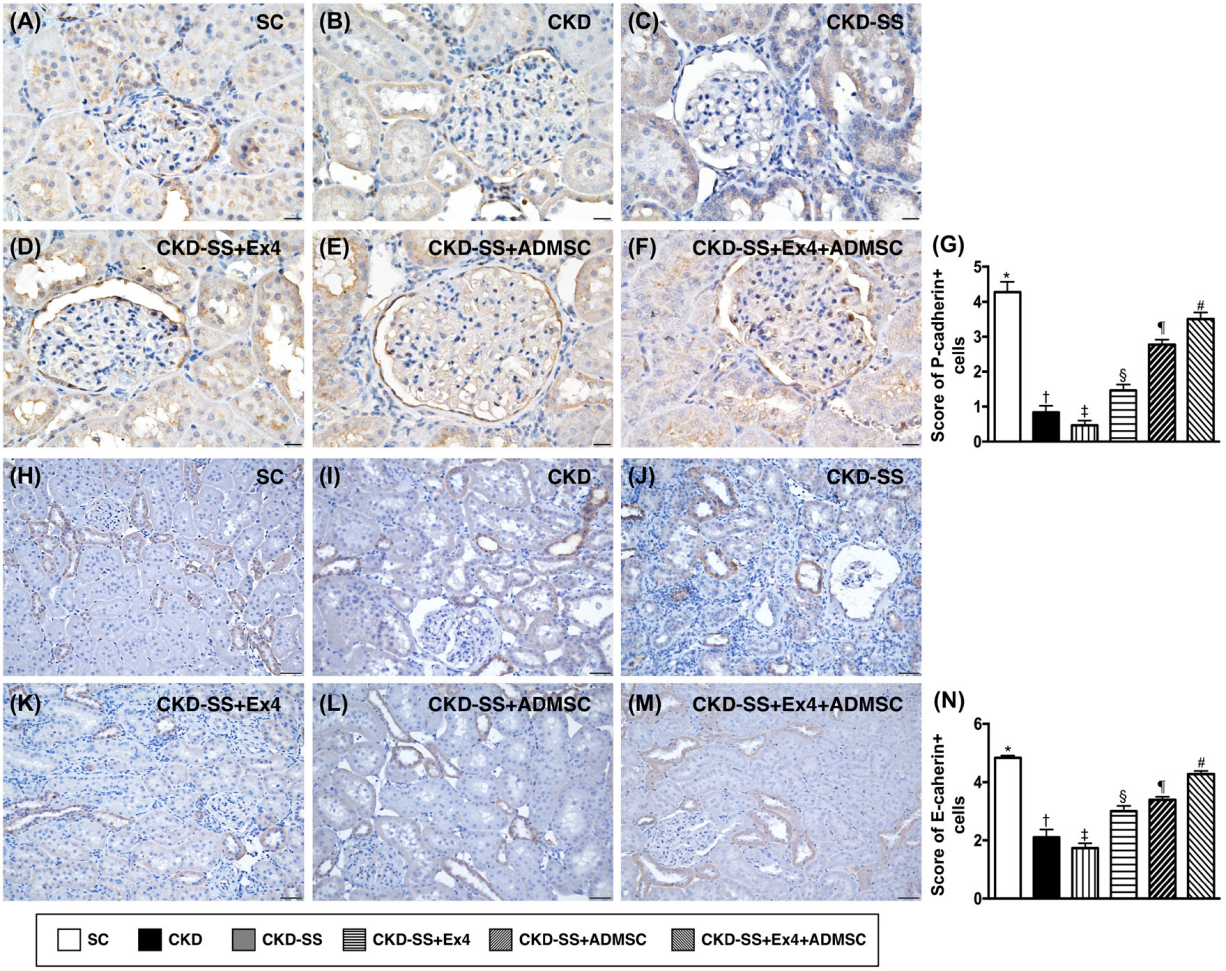
Cellular expression of P-cadherin and E-cadherin in kidney parenchyma at day 47 after CKD induction **(A** to **F)** Immunohistochemical (IHC) microscopic finding (400x) for identification of P-cadherin expression (brown color), predominantly in glomeruli. Scale bars in right lower corner represent 20μm. **(G)** Analytical results of P-cadherin expression, ^*^ vs. other groups with different symbols (†, ‡, §, ¶, ^#^), p<0.0001. **(H** to **M)** IHC microscopic finding (200x) for identification of E-cadherin expression (brown color), predominantly in renal tubular epithelial cells. Scale bars in right lower corner represent 50μm. **(N)** Analytical results of E-cadherin expression, ^*^ vs. other groups with different symbols (†, ‡, §, ¶, ^#^), p<0.0001. All statistical analyses were performed by one-way ANOVA, followed by Bonferroni multiple comparison post hoc test (n=6 for each group). Symbols (^*^, †, ‡, §, ¶, ^#^) indicate significance (at 0.05 level). SC = sham control; CKD = chronic kidney disease; SS = sepsis syndrome; ADMSC = adipose derive mesenchymal stem cell; Ex4 = exendin 4.

### Cellular expression of dystroglycan and nephrin at day 47 after CKD induction (Figure 10)

IHC microscopy revealed that the expression of dystroglycan (at the base of foot processes) and nephrin (functioning of the renal filtration barrier), two components of podocyte foot processes, were highest in group 1, lowest in group 3, significantly lower in group 2 than in groups 4 to 6, significantly lower in groups 4 and 5 than in group 6, and significantly lower in group 4 than in group 5.

**Figure 10:**
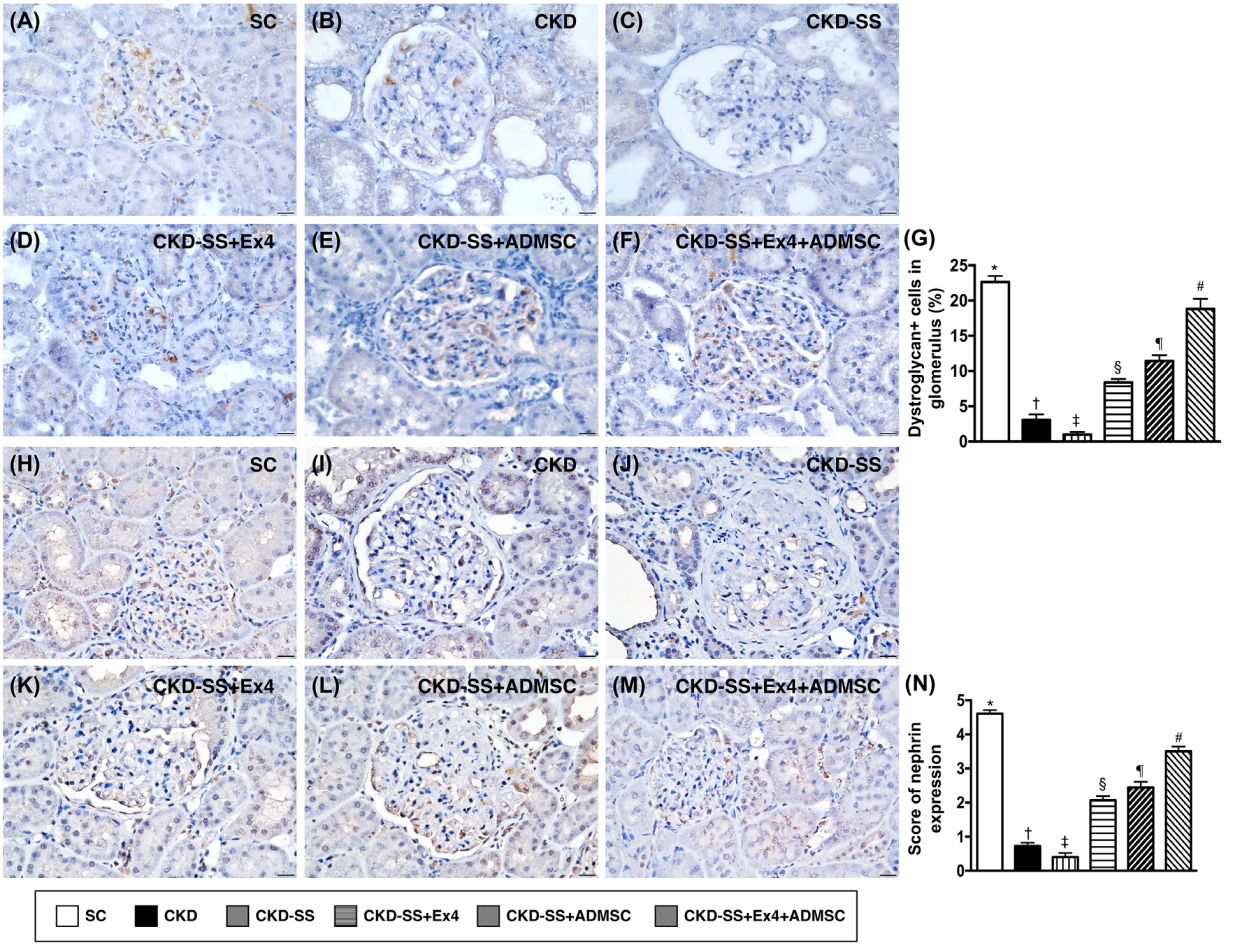
Cellular expression of dystroglycan and nephrin in kidney parenchyma at day 47 after CKD induction **(A** to **F)** Immunohistochemical (IHC) microscopic finding (400x) for identification of dystroglycan (brown color), a component of podocyte foot processes. **(G)** Analytical results of dystroglycan expression, ^*^ vs. other groups with different symbols (†, ‡, §, ¶, ^#^), p<0.0001. **(H** to **M)** IHC microscopic finding (400x) for identification of nephrin (brown color), a component of podocyte foot processes. **(N)** Analytical results of nephrin expression, ^*^ vs. other groups with different symbols (†, ‡, §, ¶, ^#^), p<0.0001. All the scale bars in right lower corner represent 20μm. All statistical analyses were performed by one-way ANOVA, followed by Bonferroni multiple comparison post hoc test (n=6 for each group). Symbols (^*^, †, ‡, §, ¶, ^#^) indicate significance (at 0.05 level). SC = sham control; CKD = chronic kidney disease; SS = sepsis syndrome; ADMSC = adipose derive mesenchymal stem cell; Ex4 = exendin 4.

### Cellular expression of fibroblast-specific protein 1 (FSP-1) and kidney injury molecule (KIM)-1 at day 47 after CKD induction (Figure 11)

IHC analysis demonstrated that change in the expression of FSP-1, predominantly situated in kidney interstitials, was lowest in group 1 and highest in group 3, significantly higher in group 2 than in groups 4 to 6, significantly higher in groups 4 and 5 than in group 6, and significantly higher in group 4 than in group 5. Additionally, the IF microscopic analysis demonstrated that change in the KIM-1, a kidney injury biomarker predominant expression in renal tubules, exhibited an identical pattern of FSP-1 among the six groups.

**Figure 11:**
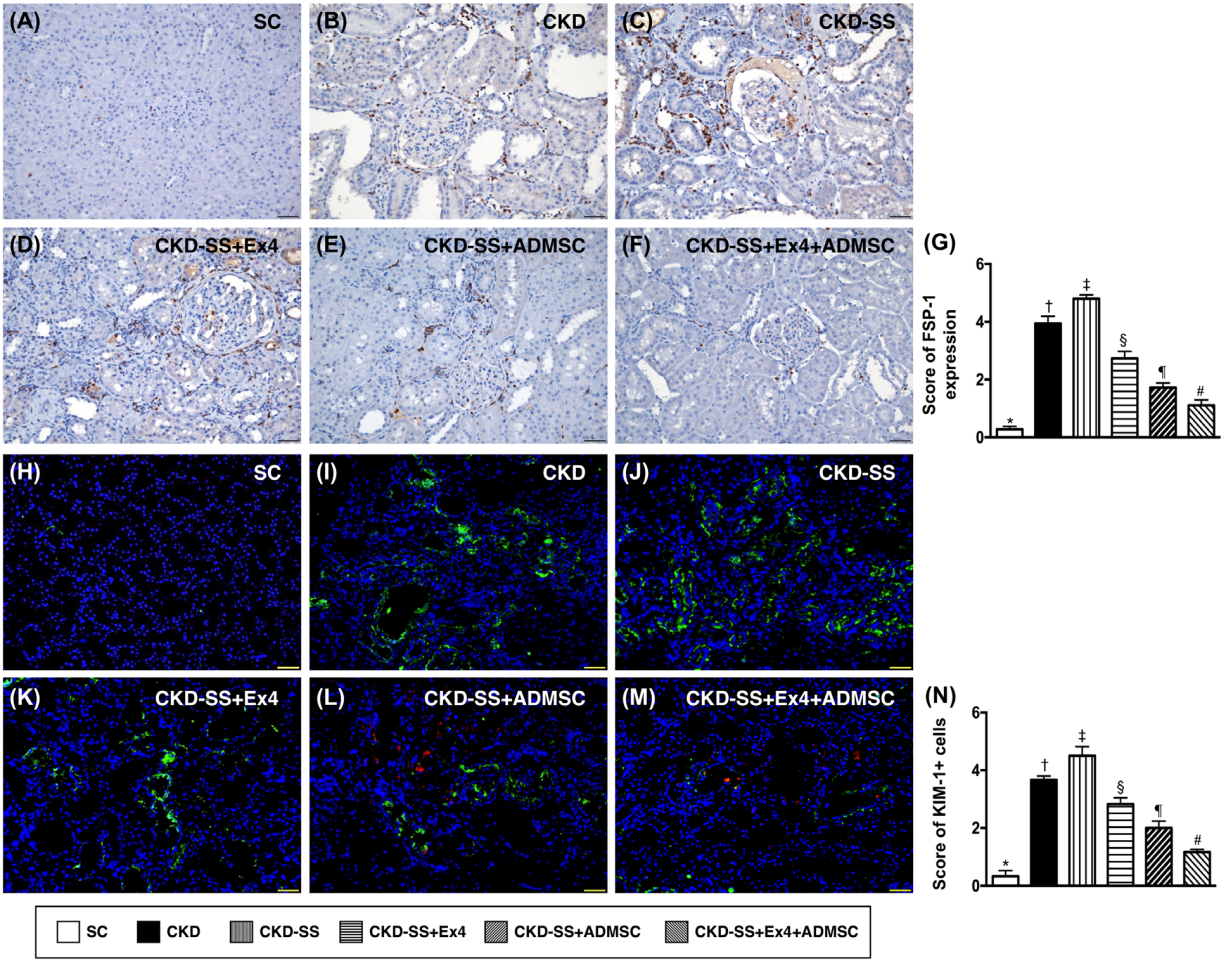
Cellular expression of fibroblast-specific protein 1 (FSP-1) and kidney injury molecule (KIM)-1 in kidney parenchyma at day 47 after CKD induction **(A** to **F)** Immunohisotochemical microscopic finding (200x) for identification of FSP-1 expression (brown color), predominantly situated in kidney interstitials. **(G)** Analytical results of FSP-1 expression, ^*^ vs. other groups with different symbols (†, ‡, §, ¶, ^#^), p<0.0001. **(H** to **M)** Immunofluorescent microscopic finding (200x) for identification of KIM-1 expression (green color), predominant expression in renal tubules. Red color in (L) and (M) indicated ADMSCs were found in kidney parenchyma. **(N)** Analytical results of KIM-1 expression, ^*^ vs. other groups with different symbols (†, ‡, §, ¶, ^#^), p<0.0001. Nuclei were stained by DAPI (blue color). All the scale bars in right lower corner represent 50μm. All statistical analyses were performed by one-way ANOVA, followed by Bonferroni multiple comparison post hoc test (n=6 for each group). Symbols (^*^, †, ‡, §, ¶, ^#^) indicate significance (at 0.05 level). SC = sham control; CKD = chronic kidney disease; SS = sepsis syndrome; ADMSC = adipose derive mesenchymal stem cell; Ex4 = exendin 4.

### Cellular expression of HO-1 at day 47 after CKD induction (Figure 12)

The IF microscopic finding demonstrated that the change in expression of HO-1, an indicator of anti-oxidant, was significantly progressively increased from group 1 to 6, suggesting an intrinsic response to ischemic/infectious stimulation and to be further enhanced after exendin-4 and ADMSC treatment.

**Figure 12:**
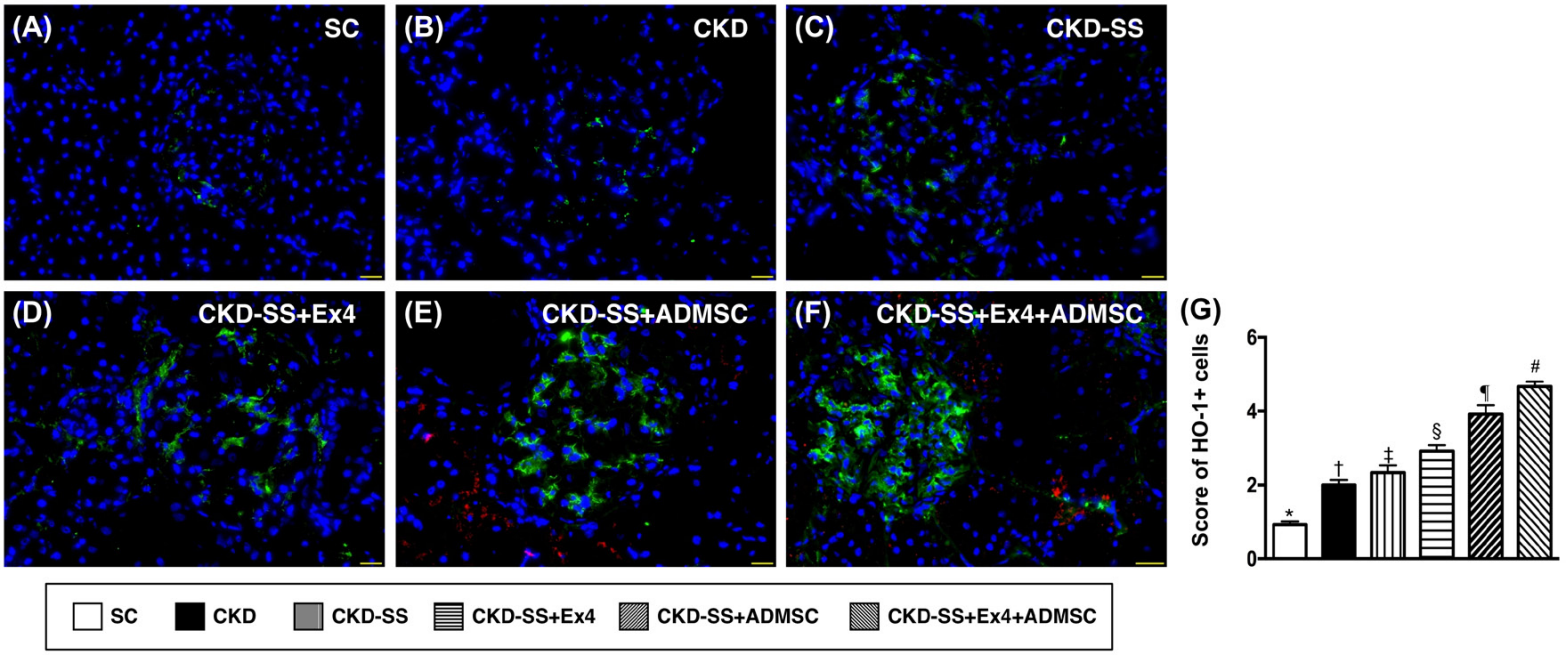
Cellular expression of anti-oxidant in parenchyma at day 47 after CKD induction **(A** to **F)** Immunofluorescent microscopic finding (400x) for identification of heme oxygen (HO)-1 (green color) expression. Nuclei were stained by DAPI (blue color). Scale bars in right lower corner represent 20μm. **(G)** Analytical results of HO-1 expression, ^*^ vs. other groups with different symbols (†, ‡, §, ¶, ^#^), p<0.0001. All statistical analyses were performed by one-way ANOVA, followed by Bonferroni multiple comparison post hoc test (n=6 for each group). Symbols (^*^, †, ‡, §, ¶, ^#^) indicate significance (at 0.05 level). SC = sham control; CKD = chronic kidney disease; SS = sepsis syndrome; ADMSC = adipose derive mesenchymal stem cell; Ex4 = exendin 4.

## DISCUSSION

In this study, we investigated the impact of Ex4-ADMSC therapy in the preservation of residual renal function in coexisting CKD-SS. The study has several implications for our future clinical practice. First, we successfully created an experimental model of CKD-SS to investigate the underlying mechanism of SS to deteriorate the residual renal function in the setting of CKD. Second, the results of the present study showed that the underlying mechanism of SS involving in the deteriorating residual renal function of CKD is multi-factorial, highlighting that drug combination therapy is superior to a single therapy for improving the prognostic outcome in the CKD-SS setting. Third, we found that combined Ex4-ADMS therapy is superior to either one alone in attenuating the molecular-cellular perturbations in kidney parenchyma and preserving the residual renal function in the CKD-SS setting.

One important finding in the present study was that the circulating levels of creatinine and BUN (i.e., indices of functional kidney) at the end of study period were notably higher in the CKD-SS group than in the CKD group, suggesting that residual renal function is deteriorated by SS. The cause of this event has been identified to be a strongly independent predictor of poor prognostic outcome in CKD patients [19, 20].

The most important findings in the present study are that the collagen deposition area (i.e., indicated kidney fibrosis) and histopathological finding of kidney injury score (an anatomical expression) were markedly increased in CKD animals as compared with in SC animals. Interestingly, previous studies have revealed that fibrosis is the unifying pathway leading to CKD [29, 30]. Our finding is supported by previous studies [29, 31]. Of particular importance, compared with CKD group, fibrosis was significantly increased in the CKD-SS group. Our finding, in addition to partially explaining why the kidney injury score was much higher in CKD group than in SC group, highlights the key role of SS in worsening the residual kidney function.

The link between the CKD setting and upregulation of apoptosis, oxidative stress, mitochondrial/DNA damage, and inflammatory reaction have been well recognized by previous experimental studies [22, 29] as well as by a clinical trial [30]. Another important finding in the present study was that the levels of apoptosis at the protein and cellular levels were substantially higher in CKD-SS group as compared with the CKD only group. Additionally, the inflammatory, oxidative stress and DNA damage biomarkers were remarkably higher, whereas the endothelial integrity biomarker (i.e., eNOS) was notably lower in CKD-SS animals than in CKD animals. In this way, our findings, in addition to strengthening the findings of previous studies [22, 29, 30], can at least in part, explain why the kidney function and kidney injury score were significantly higher in CKD animals and more significantly higher in CKD-SS animals.

It is well-known that the integrity of podocytes and the components of podocyte foot processes is an important barrier for preventing proteinuria. Our previous experimental study showed that the rat glomerular and renal tubular architectures were inevitably damaged by acute kidney ischemia-reperfusion injury [21]. Therefore, the ratio of urine protein-to-creatinine was found to be markedly increased in these animals after acute ischemia-reperfusion injury [21]. A principal finding in the present study was that the ratio of urine protein to creatinine was significantly increased in CKD and more significantly increased in CKD-SS animals than in the SC animals. Additionally, not only the protein expression but also the microscopy findings of the renal ultrastructural integrity of glomeruli (i.e., the components of podocyte foot processes) were remarkably deteriorated in CKD and more remarkably deteriorated in CKD-SS animals than in those of SC animals. These findings, in addition to being comparable with our previous study [21], may explain why the proteinuria was the most severe in CKD-SS animals.

Our studies previously identified that Ex4 and ADMSC therapy significantly protected kidney architecture and renal function from sepsis-induced or ischemia-reperfusion kidney injury [21, 22, 24, 25, 28]. A particularly important finding in the present study was that Ex4 was comparable to ADMSC therapy in protecting the residual renal function from CKD-SS and combined Ex4-ADMSC was superior to either one alone for protecting kidney against CDK-SS injury. In this way, our findings corroborated with the findings of previous studies [21, 22, 24, 25, 28], suggesting that this combined therapy may have potential for patients in the clinical setting of CKD-SS, especially for diabetic patients who suffer from CKD-SS and are refractory to conventional therapy.

The mortality rate was not originally designed to investigate in the present study. Intriguingly, our recent study [26] has shown that the mortality rate was significantly lower in urogenital-organ SS rats with than in without ADMSC-ciprofloxacin treatment. Based on the disease entity and treatment strategy was similar between in the present and our recent [26] studies, perhaps, the mortality rate was markedly reduced in rats after receiving ADMSC-ciprofloxacin treatment in our recent study [26] is also reasonably predicted in our present study in rats after receiving ADMSC-Ex4 treatment.

In our daily clinical practice, we find that the prevalence of SS is notably increased in DM than in without DM patients. Additionally, MSCs with anti-inflammation and immunomodulation capacity has been proved by clinical trials to be safe and efficacious for treatment of ischemia-related organ dysfunction. Accordingly, our preclinical relevant data may raise the need of consideration of a prospective study to assess the therapeutic potential for CKD-SS patients who are refractory to conventional therapy, especially in those of DM patients.

### Study limitations

**Figure 13:**
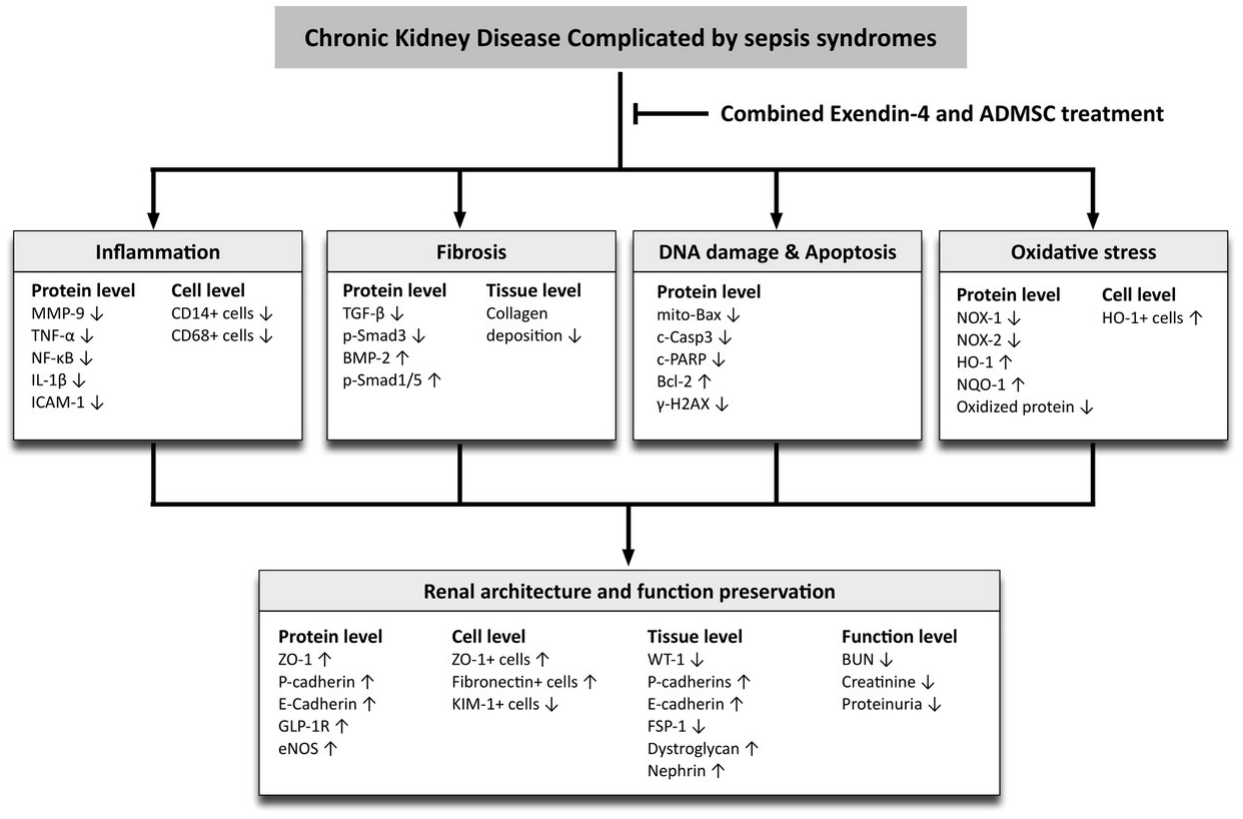
Proposed mechanisms underlying the positive therapeutic effects of combined exendin-4 and allogenic adipose-derived mesenchymal stem cell preserved renal function in a chronic kidney disease and sepsis syndrome MMP-9 = matrix metalloproteinase 9; TNF-α = tumor necrosis factor alpha; NF-κB = nuclear factor kappa B; IL-1ß = interleukin-1ß; ICAM-1 = intercellular adhesion molecule 1; TGF-ß = transforming growth factor-ß; BMP = bone morphogenetic protein; mito = mitochondrial; c-Casp 3 = cleaved caspase 3; c-PARP = protein expression of cleaved poly (ADP-ribose) polymerase; HO-1 = heme oxygenase 1; GLP-1R = glucagon like peptide 1 receptor; eNOS = endothelial nitric oxide synthase; KIM-1 = kidney injury molecule 1; WT-1 = Wilm's tumor suppressor gene 1; FSP-1 = fibroblast-specific protein 1; BUN = blood urea nitrogen.

This study has several limitations. First, the exact underlying mechanism through which Ex4-ADMSC therapy protects the residual renal function from acute CKD-SS injury is still not so clear. Our results showed that the mechanism involved is multi-factorial, including suppression of the molecular-cellular perturbations (i.e., inflammation, oxidative stress, fibrosis, apoptosis and DNA damage) and involves upregulation of several protecting factors (i.e., anti-oxidant, anti-fibrotic, anti-apoptotic and GLP-1) rather than only a single one. The proposed mechanisms of Ex4-ADMSC therapy for preserving kidney architecture and residual renal function in the setting of CKD-SS are schematically presented in Figure 13. Second, the time interval between treatment and euthanizing animals was only five days (i.e., mimicking the clinical setting of the acute phase of SS), thus, no long-term outcome is provided by the present study. Third, because the mortality rate was not in the scope of the study, so this study did not provide this information.

In conclusion, the results of the present study showed that Ex4-ADMSC therapy is superior to either therapy alone for preservation of residual renal function and architecture of kidney in the CKD-SS setting.

## MATERIALS AND METHODS

### Ethics

All animal experimental protocols and procedures were approved by the Institute of Animal Care and Use Committee at Kaohsiung Chang Gung Memorial Hospital (Affidavit of Approval of Animal Use Protocol No. 2015052702) and performed in accordance with the Guide for the Care and Use of Laboratory Animals [The Eighth Edition of the Guide for the Care and Use of Laboratory Animals (NRC 2011)].

Animals were housed in an Association for Assessment and Accreditation of Laboratory Animal Care International (AAALAC)-approved animal facility in our hospital (IACUC protocol no. 101008) with controlled temperature and light cycles (24°C and 12/12 light cycles).

### Isolation of allogenic ADMSCs

For preparation of ADMSCs (i.e., allogenic ADMSCs), an additional sixteen SD rats were used in the current study. The procedure and protocol of ADMSC preparation has been described in detail in our previous reports [24–26]. Briefly, adipose tissue surrounding the epididymis was carefully dissected, excised and prepared. Then, 200-300 μL of sterile saline was added to every 0.5 g of adipose tissue to prevent dehydration. The tissue was cut into < 1 mm^3^ size pieces using a pair of sharp, sterile surgical scissors. Sterile saline (37°C) was added to the homogenized adipose tissue in a ratio of 3:1 (saline: adipose tissue), followed by the addition of stock collagenase solution to a final concentration of 0.5 units/mL. The centrifuge tubes with the contents were placed and secured on a Thermaline shaker and incubated with constant agitation for 60 ± 15 minutes at 37°C. After step-by-step preparation [24–26], the cells were resuspended in saline. An aliquot of cell suspension was then removed for cell culture in Dulbecco’s modified Eagle’s medium (DMEM)-low glucose medium containing 10% FBS for 14 days. Approximately 2-3 × 10^6^ ADMSCs were obtained from each rat. Thirty minutes prior to the transplantation procedure, the AMDSCs were labeled with dye (Cell tracker; Molecular probes: REF: C34551) for the purpose of identification of AMDSCs in kidney parenchyma using immunofluorescent (IF) microscopic examination.

### Cell Tracker prepare and procedure and protocol of ADMSC labeling

The Cell Tracker dye was first centrifugated for the powder spin down, followed by added 1 mL without phenol-red DMEM medium to make the concentration as 1.0 μM and stored at -20. The protocol of Cell Tracker staining at step by step as shown at the following:

1. Trypsinized the cells and then washed twice with PBS;
2. Resuspended the cells (10^6^-10^7^ cells) in 200 μl without phenol-red DMEM medium;
3. Added Cell Tracker 5 μl (stock Cell Tracker = 1 μM for the cells staining; the cell tracker concentration ranged in 5 nM-25 nM)
4. Incubated at 37 for 30-60 minutes;
5. Finally, the cells were resuspended in desired volume and ready for transfusion.

### Preparation of abdominally-derived bacteria using cecal ligation and puncture (CLP) for induction of sepsis syndrome

At day 42 after CKD induction SS was induced. The procedure for preparing abdominally-derived bacteria was described in our previous report [26]. Briefly, five additional SD rats were anesthetized by inhalational of 2.0% isoflurane and placed in a supine position on a warming pad at 37°C with the abdomen shaved. Under sterile conditions, the abdominal skin and muscle were opened and the cecum exposed. In the experimental CLP animals, the cecum was ligated by prolene over its distal portion (i.e., distal ligation) and the cecum distal to the ligature was punctured twice with an 18G needle to allow the cecal contents to be expressed intra-peritoneally, as previously described [27, 28]. The abdominal wound was closed and the animal was allowed to recover from anesthesia. Thirty-six hours after the CLP procedure, the abdomens of the five animals were opened again and the ascites which contained colon-derived bacteria (mixed bacteria) were collected and pooled for intraperitoneal injection in the experimental groups except for the sham controls. The ascites were collected for quantitative analysis of the number of bacteria by counting bacteria colonies in a high-power field with Gram stain.

### Animal model of CKD

The procedure and protocol of CKD induction have been described in our previous reports [22, 29]. In detail, pathogen-free, adult male Sprague-Dawley (SD) rats (n=48) weighing 320-350 g (Charles River Technology, BioLASCO Taiwan Co. Ltd., Taiwan) were used in the present study. All animals were anesthetized by inhalational of 2.0% isoflurane, placed in a supine position on a warming pad at 37°C for midline laparotomies. The SC group received laparotomy only, while CKD was induced in other groups of animals by right nephrectomy plus arterial ligation of upper and middle thirds of blood supplies to the left kidney. Such a model allows preservation of a limited amount of functioning renal parenchyma to simulate the condition of CKD.

### Animal grouping and rationale of utilizing dosages of bacteria, ADMSC and exendin-4 in the present study

Animals (n=36) were randomly and equally divided into six groups (i.e., eight animals each): group 1 (SC), group 2 (CKD), group 3 [CKD-SS (by intra-peritoneal injection of cecal ligation puncture (CLP)-derived bacteria only (1.0 × 10^4^ mixed bacteria/mL, total 5.0 mL abdominal fluid per rat)], group 4 (CKD-SS + exendin-4 (Ex4) (10 μg/kg/day by intra-peritoneal injection 30 min and at days 1 to 5 since after SS induction), group 5 [CKD-SS + ADMSCs (2.0 × 10^6^ cells was intravenously administered at 30 min after SS induction)], and group 6 (CKD-SS + Ex4 + ADMSCs).

The regimen of exendin-4 to be administered for the animals was also based on our recent reports [21–23]. Additionally, the therapeutic dosage of ADMSCs was based on our previous reports [24–28] with minimal modification. Furthermore, the dosage of abdominally-derived bacteria to be utilized for induction of SS in the current study was based on our recent report [26].

### Histopathology scoring of CKD at day 5 after SS induction

Histopathology scoring was determined in a blinded fashion as previously reported [22, 29]. In brief, the kidney specimens from all animals were fixed in 10% buffered formalin, embedded in paraffin, sectioned at 5 μm and stained with hematoxylin and eosin (H & E) for light microscopy. The scoring system reflecting the grading of tubular necrosis, loss of brush border, cast formation, and tubular dilatation in 10 randomly chosen, non-overlapping fields (200×) for each animal was as follows: 0 (none), 1 (≤10%), 2 (11–25%), 3 (26–45%), 4 (46–75%), and 5 (≥76%).

### Assessment of serum creatinine and BUN levels, and collection of 24-hour urine for the ratio of urine protein to creatinine

Blood samples were collected from all animals in each group to assess changes inserum creatinine and blood urine nitrogen (BUN) levels at baseline and at days 35 and 47 after CKD induction. Quantification of BUN and creatinine level was performed using standard laboratory equipment at our hospital.

The procedure and protocol for collection of 24-h urine for determining the ratio urine to creatinine was based on our previous report [21]. In details, each animal was put into a metabolic cage [DXL-D, space: 190 x 290 x 550, Suzhou Fengshi Laboratory Animal Equipment Co. Ltd., Mainland China] for 24 hrs with free access to food and water. Urine in 24 hrs was collected in all animals at baseline and at days 35 and 47 after the CKD induction procedure.

### Immunohistochemical (IHC) and immunofluorescent (IF) staining

The procedure and protocol of IF staining have been described in details in our previous reports [24–28]. For IHC and IF staining, rehydrated paraffin sections were first treated with 3% H_2_O_2_ for 30 minutes and incubated with Immuno-Block reagent (BioSB, Santa Barbara, CA, USA) for 30 minutes at room temperature. Sections were then incubated with primary antibodies specifically against zonula occludens-1 (ZO-1) (1:300, Abcam, Cambridge, MA, USA), Wilm's tumor suppressor gene 1 (WT-1) (1:1000, Abcam, Cambridge, MA, USA), kidney injury molecule (KIM)-1 (1:200, R&D system, Minneapolis, MN, USA), fibroblast specific protein (FSP)-1 (1:200, Abcam, Cambridge, MA, USA), P-cadherin (1:100, Novus, Littleton, CO, USA), E-cadherin (1:400, Abcam, Cambridge, MA, USA), fibronectin (1:200, Abcam, Cambridge, MA, USA), synaptopodin (1:500, Santa Cruz, Santa Cruze, CA, USA), nephrin (1:200, Bioss, Massachusetts, Boston, USA), Sirius red (1% sirius red in saturated picric acid solution), CD14 (1:200, Santa Cruz Biotechnology, CA, USA), CD68 (1:100, Abcam, Cambridge, MA, USA) and heme oxygenase (HO)-1 (1:250, Abcam, Cambridge, MA, USA), while sections incubated with the use of irrelevant antibodies served as controls. Three sections of kidney specimen from each rat were analyzed. For quantification, three randomly selected HPFs (200x or 400x for IHC and IF studies) were analyzed in each section. The mean number of positively-stained cells per HPF for each animal was then determined by summation of all numbers divided by 9.

An IHC- and IF-based scoring system was adopted for semi-quantitative analyses of all the IHC-stained biomarkers and the IF-stained HO-1 in the kidney as a percentage of positive cells in blinded fashion (score of positively-stained cells: 0 = negative staining; 1= <15%; 2 = 15-25%; 3 = 25-50%; 4 = 50-75%; 5= >75%-100%/per HPF). Additionally, the expressions of WT-1 and dystroglycan were calculated as the number of positively-stained cells in podocytes divided by total cells in glomerular tuft and were expressed as % by analytical results in each group.

### Western blot analysis

The procedure and protocol for Western blot analysis were based on our recent reports [24–28]. Briefly, equal amounts (50 mg) of protein extracts were loaded and separated by SDS-PAGE using acrylamide gradients. After electrophoresis, the separated proteins were transferred electrophoretically to a polyvinylidene difluoride (PVDF) membrane (Amersham Biosciences, Amersham, UK). Nonspecific sites were blocked by incubation of the membrane in blocking buffer [5% nonfat dry milk in T-TBS (TBS containing 0.05% Tween 20)] overnight. The membranes were incubated with the indicated primary antibodies [matrix metalloproteinase (MMP)-9 (1:3000, Abcam, Cambridge, MA, USA), tumor necrosis factor (TNF)-α (1:1000, Cell Signaling, Danvers, MA, USA), nuclear factor (NF)-κB (1:600, Abcam, Cambridge, MA, USA), interleukin (IL)-1ß (1:1000, Cell Signaling, Danvers, MA, USA), intercellular adhesion molecule (ICAM)-1 (1:1000, Abcam, Cambridge, MA, USA), NOX-1 (1:1500, Sigma, St. Louis, Mo, USA), NOX-2 (1:750, Sigma, St. Louis, Mo, USA), mitochondrial Bax (1:1000, Abcam, Cambridge, MA, USA), cleaved (c) caspase (c-Casp 3) (1:1000, Cell Signaling, Danvers, MA, USA), cleaved poly (ADP-ribose) polymerase (c-PARP) (1:1000, Cell Signaling, Danvers, MA, USA), Bcl-2 (1:600, Abcam, Cambridge, MA, USA), endothelial nitric oxide synthase (eNOS) (1:1000, Abcam, Cambridge, MA, USA), phosphorylated (p)-Smad3 (1:1000, Cell Signaling, Danvers, MA, USA), p-Smad1/5 (1:1000, Cell Signaling, Danvers, MA, USA), transforming growth factor (TGF)-1ß (1:5000, Abcam, Cambridge, MA, USA), bone morphogenetic protein (BMP)-2 (1:500, Abcam, Cambridge, MA, USA), zonula occludens-1 (ZO-1) (1:1000, Abcam, Cambridge, MA, USA), NAD (P) H quinone dehydrogenase (NQO1) (1:1000, Abcam, Cambridge, MA, USA), P-cadherin (1:1000, Abcam, Cambridge, MA, USA), E-cadherin (1:1000, Abcam, Cambridge, MA, USA), HO-1 (1:500, San Diego, CA United States), glucagon like peptide-1 receptor (GLP)-1R (1:1000, Abcam, Cambridge, MA, USA) and actin (1: 10000, Chemicon, Billerica, MA, USA)] for 1 hour at room temperature. Horseradish peroxidase-conjugated anti-rabbit immunoglobulin IgG (1:2000, Cell Signaling, Danvers, MA, USA) was used as a secondary antibody for one-hour incubation at room temperature. The washing procedure was repeated eight times within one hour. Immunoreactive bands were visualized by enhanced chemiluminescence (ECL; Amersham Biosciences, Amersham, UK) and exposed to Biomax L film (Kodak, Rochester, NY, USA). For the purpose of quantification, ECL signals were digitized using Labwork software (UVP, Waltham, MA, USA).

### Assessment of oxidative stress

The procedure and protocol for assessing the protein expression of oxidative stress have been described in details in our previous reports [24–28]. The Oxyblot Oxidized Protein Detection Kit was purchased from Chemicon, Billerica, MA, USA (S7150). DNPH derivatization was carried out on 6 μg of protein for 15 minutes according to the manufacturer’s instructions. One-dimensional electrophoresis was carried out on 12% SDS/polyacrylamide gel after DNPH derivatization. Proteins were transferred to nitrocellulose membranes which were then incubated in the primary antibody solution (anti-DNP 1: 150) for 2 hours, followed by incubation in secondary antibody solution (1:300) for 1 hour at room temperature. The washing procedure was repeated eight times within 40 minutes. Immunoreactive bands were visualized by enhanced chemiluminescence (ECL; Amersham Biosciences, Amersham, UK) which was then exposed to Biomax L film (Kodak, Rochester, NY, USA). For quantification, ECL signals were digitized using Labwork software (UVP, Waltham, MA, USA). For oxyblot protein analysis, a standard control was loaded on each gel.

### Statistical analysis

Quantitative data are expressed as means ± SD. Statistical analysis was performed by ANOVA followed by Bonferroni multiple-comparison post hoc test. SAS statistical software for Windows version 8.2 (SAS institute, Cary, NC) was utilized. A probability value of less than 0.05 was considered statistically significant.
